# PD48 Dosing Variability Of Anti-Scorpionic Serum In Brazil: A Call For Standardization

**DOI:** 10.1017/S0266462325101815

**Published:** 2025-12-29

**Authors:** Amanda das Dores, Stéfani Borges, Bruna Santos, Daniela Melo

## Abstract

**Introduction:**

Accidents involving scorpions of the *Tityus* genus are common in Brazil. While there is no consensus on the ideal dose of anti-scorpionic serum, its use is not recommended for mild cases, whereas two to four vials are suggested for moderate cases and four to six vials for severe cases. Our objective was to synthesize the available literature on serum dosage in Brazil.

**Methods:**

A scoping review was conducted of Brazilian studies on patients with suspected or confirmed scorpion envenomation that described serum dosages used for treatment (protocol registered on Open Science Framework DOI 10.17605/OSF.IO/8F7ZB). The study followed the PRISMA Extension for Scoping Reviews guidelines. The ROBINS-I tool was used for risk of bias assessment, with a traffic light plot generated using the Robvis app. Data collection and analysis were performed by two independent reviewers, with a third reviewer consulted when necessary.

**Results:**

After screening 410 records, five studies were included. One study reported using 7.5 (standard deviation [SD] 3.5) vials for mild cases, 4.0 (SD 1.7) for moderate, and 6.5 (SD 2.1) for severe cases. Another study described one to 10 vials for mild cases, one to 15 for moderate, and one to 20 for severe cases. Other studies report using one to six vials, four vials, or that most patients received no more than four vials, although none of these studies provided the risk classification. All studies presented a high risk of bias, mainly due to lack of control for confounding variables.

**Conclusions:**

Available data on anti-scorpionic serum use in Brazil revealed significant variability in clinical management, with the volumes administered often exceeding the Ministry of Health’s recommendations. This highlights a lack of standardization, which increases the likelihood of resource waste in the health care system and safety concerns. Strategies such as educational initiatives and clinical audits are recommended to promote rational serum use.

